# You’ll be a clinician-scientist, my son

**DOI:** 10.1186/s12967-015-0713-8

**Published:** 2015-11-04

**Authors:** Pierre R. Smeesters

**Affiliations:** Group A Streptococcus Research Group, Murdoch Childrens Research Institute, Melbourne, Australia; Centre for International Child Health, University of Melbourne, Melbourne, Australia; Department of Paediatrics, Hôpital Universitaire des Enfants Reine Fabiola, Brussels, Belgium; Molecular Bacteriology Laboratory, Université Libre de Bruxelles, Brussels, Belgium

## Abstract

Opinion-based commentary about the complex reality of being a clinician-scientist in today’s modern biomedical environment. The essay uses the beautiful, but old, poem “If” from Rudyard Kipling to draw a parallel with the ambitions, dreams and limits of being a clinical-scientist today.

Rudyard Kipling beautiful poem ‘If’ starts with the following verses:

If you can keep your head when all about you

    Are losing theirs and blaming it on you;  

…

And yet don’t look too good, nor talk too wise;

As practitioners, we learn to act during times of acute or prolonged crisis. We try hard to make ourselves strong enough to take responsibility for our actions and provide solutions to patients. Medical training is exigent; so too is the clinical practice which is also very dynamic in nature, with rapid rewards or as rapid disappointments. Whilst aspects of medical practice can sometimes be seen as repetitive, facing the human condition in all its magnificence and weakness is assuredly an inspiring privilege. Although aiming for a cure, practitioners see the limitation of the so-called ‘art of curing’ on a daily basis. If practitioners agree to recognise it, humility is waiting for you at the corner of your practice. Practitioners try to reach the best equilibrium point possible for any given patient. The seemingly ‘better’ or the purported ‘perfect’ is in reality not always the ‘good’ for a particular patient. Doctors therefore listen, observe and finally propose, with a mixture of both cutting-edge knowledge and common sense, a balanced strategy that will usually help the patient in overcoming their medical issue.

Researchers rather desperately look for the disequilibrium point, the scientific breakthrough. On the brink of what is known, we have the intoxicating mission of pushing the scientific community in the direction our results suggest. Exploring the unknown is fascinating, exciting and a source of real pleasure. Of course, science cannot evolve without open debate, and that is sometimes accompanied by some vehemence in the argument or in the communication. Strong—a scientist must therefore certainly become as well. Scientists’ expectations are very high and good research projects are developed with ambition, intuition and vision. Researchers listen to the evidence obtained and use it to develop, and fund, the next research project. Repetition is not often fruitful and renewed creativity is a must. Patience, however, is necessary since the most important projects run over many years. It is fair to say that humility, although still elegant at the personal level, is not a mandatory quality to become a successful scientist. Today’s science is notably assessed through cheerleader endorsement, and cultivating popularity, if not self-sufficient, is also necessary for success.

If you can dream—and not make dreams your master;

    If you can think—and not make thoughts your aim;

If you can meet with Triumph and Disaster

    And treat those two impostors just the same;

In both medical practice and scientific research, we have to travel back and forth from dream to reality. If this travel is sometimes tiresome, it is also extremely rich in opportunities. Much can be learned from both ‘Triumph’ and ‘Disaster’. There is, surprisingly, much life in death and successes always are so relative. Time moves rapidly and both successes and failures soon belong to the past. To some extent, acting as a practitioner or a researcher can teach us not to become (too) enslaved to our successes or (too) guilty of our failures. Reconciliation of the opposite is certainly, as in Kipling’s prose, a common lesson we can learn from both appointments.

It is unsurprising that practitioners always have been involved with biomedical progress. Research is everyday and essential in medical practice, particularly for treating patients with rare conditions. To organize, expand and systematize this research activity appears therefore like a natural extension of clinical practice. Moreover, as treating practitioners, doctors have a particular profile for influencing the research agenda. There is little doubt that the position of clinician-scientist appears to be distinctive, necessary and important. A clinician-scientist can provide a unique vision about the contribution that modern science can bring to update medical care. Additionally, both the clinical and scientific working environments, although diverse, are synergistic in nature. Appointing individuals demonstrating some level of expertise in both areas is certainly an interesting opportunity for cross-fertilisation between them.

Undoubtedly, having worked as a scientist has opened new horizons to my clinical practice. Experiencing how little we really know about disease and how vast are the unanswered questions has increased my ability to listen to patients and make them participating members of any decision taken. The less we know, the more room for the patient’s own vision about their condition. This does not mean that each medical option is equally efficient but simply represents a more integrated assessment about the solution each option can provide. In today’s medicine, patients want to be considered as partners and key deciders for their future, and this involvement represents a fantastic and very positive evolution of medical care.

Performing science has also helped me to understand how limited my area of expertise was. We are usually pleased to believe that we know a great deal but experimental science consistently demonstrates how restricted our real understanding is. A natural reaction after facing this ascertainment is to favour multidisciplinary collaborations to solve any given medical problem.

On the other hand, my medical background has certainly been instrumental in all of my research projects. Firstly, the medical vision has influenced the kind of project I decided to be involved with. I also like to believe that the medical context of each research project has favoured the ongoing interest of research staff and students, probably by allowing them a better understanding of the overall picture of a project and the motives to develop specific technical expertise in the long term. This preserved enthusiasm has been a key factor of successful projects completion.

If you can make one heap of all your winnings

    And risk it on one turn of pitch-and-toss,

And lose, and start again at your beginnings

    And never breathe a word about your loss;

However truly inspiring these verses might be, I cannot read them without adding a ‘but’ to the ‘if’. If determination is certainly a necessary quality for acting as a practitioner or a scientist, the line between stoicism and resistance to change can be thin. And so is the frontier between heroism and stupidity. The reading of this poem touches my soul but it possesses a subtly antiquated and withdrawn sentiment that to me does ring odd. As a clinician, I seriously doubt that forsaking the possibility to converse about our losses constitutes the best option to recover from them. As a scientist, I painfully doubt that it is still possible to lose everything and start again at your beginnings…

Refusing to see how the scientific potential of the clinician-scientist has evolved in today’s science does not elevate the debate. Clinician-scientists no longer drive biomedical research. It is not possible to be truly proficient in both modern clinical care and experimental basic science. In addition, and because they rarely elucidate the latest biological mechanism, their research output will not always be considered as they would have wished by some basic scientists and top tier scientific journals. The constraints of the daily routine of medical practice, including the increasing financial pressure on the health system, lack of time and even the lack of training are major obstacles to the development of broader research activity within academic teaching hospitals. Even if medical training provides us with some scientific knowledge and maybe even more importantly with the interest to learn more about science, it does not make us experimental scientists in and of itself. And if a practitioner decides to devote time to acquire some experience as an experimental scientist, it will happen at the price of their clinical experience and practice.

As alluded to in Kipling’s prose, the clinician-scientist should also consider carefully the blinding effect of money. If one of the conditions for scientific power is constituted by the access to resources, it would certainly be sad not to use it wisely. To mirror the evolution in social standing that practitioners have had to face over recent decades, clinician-scientists may be asked to modernise their role in scientific discoveries. Changes often also carry opportunities for improvements and certainly do not mean any renouncement to the ideal driving this incredible multidisciplinary position.

If neither foes nor loving friends can hurt you,

…

Yours is the Earth and everything that’s in it,

    And - which is more - you’ll be a Man, my son!

Who can really define what to be a clinician-scientist means today? There are as many definitions as there are people embracing this career. Being a clinician-scientist is an incredible position offering many opportunities to construct a multidisciplinary team working on carefully designed research projects addressing patient-oriented problems. Clinician-scientists possess a unique vision about science and can certainly develop research as a means of fulfilment in their work, as well as training in rigor and critical thinking. Clinician-scientists can be, and must remain, knowledge brokers or bridge builders. In our highly specialized medical and research modern environment, they possess an interesting and much needed profile allowing them to make connections between people and expertise. This specific role may be even more important in the future. Of course, such sustained research activity cannot be conceived without full academic freedom and this kind of freedom is rarely given by any institutional power but must rather be taken at all levels—practically, intellectually and financially.

Will I thus really be a clinician-scientist dad? Well, maybe in some aspects and probably in some peoples’ opinion, but with the personal feeling of certainly being a bit of both but definitely not all of each. But that’s absolutely fine, it is fascinating and I like it!

